# Construction of a radiation hybrid map of chicken chromosome 2 and alignment to the chicken draft sequence

**DOI:** 10.1186/1471-2164-6-12

**Published:** 2005-02-04

**Authors:** Sophie Leroux, Mélanie Dottax, Suzanne Bardes, Florence Vignoles, Katia Fève, Frédérique Pitel, Mireille Morisson, Alain Vignal

**Affiliations:** 1 Laboratoire de Génétique Cellulaire, INRA, Castanet-Tolosan, 31326, France

## Abstract

**Background:**

The ChickRH6 whole chicken genome radiation hybrid (RH) panel recently produced has already been used to build radiation hybrid maps for several chromosomes, generating comparative maps with the human and mouse genomes and suggesting improvements to the chicken draft sequence assembly. Here we present the construction of a RH map of chicken chromosome 2. Markers from the genetic map were used for alignment to the existing GGA2 (Gallus gallus chromosome 2) linkage group and EST were used to provide valuable comparative mapping information. Finally, all markers from the RH map were localised on the chicken draft sequence assembly to check for eventual discordances.

**Results:**

Eighty eight microsatellite markers, 10 genes and 219 EST were selected from the genetic map or on the basis of available comparative mapping information. Out of these 317 markers, 270 gave reliable amplifications on the radiation hybrid panel and 198 were effectively assigned to GGA2. The final RH map is 2794 cR_6000 _long and is composed of 86 framework markers distributed in 5 groups. Conservation of synteny was found between GGA2 and eight human chromosomes, with segments of conserved gene order of varying lengths.

**Conclusion:**

We obtained a radiation hybrid map of chicken chromosome 2. Comparison to the human genome indicated that most of the 8 groups of conserved synteny studied underwent internal rearrangements. The alignment of our RH map to the first draft of the chicken genome sequence assembly revealed a good agreement between both sets of data, indicative of a low error rate.

## Background

Chicken is a model organism in various fields of biology, such as embryology or immunology. It is also the only bird species for which the genome has been studied in detail and a lot is expected from its use in comparative genome analyses. This will help to detect sequences conserved between species, which should correspond to unknown exons and to regulatory or other functional regions. Such analyses will therefore be essential for the annotation of other genomes, including that of human [1,2]. Chicken is also actually the only major agricultural species for which a draft assembly of the genome sequence is available [3,4]. Thanks to a significantly lower rate of interspersed repetitive elements and to the use of a highly inbred bird for sequencing, this draft is probably more accurate than the first one published for human three years ago [5,6]. Nevertheless, previous comparisons of RH mapping data with the sequence data showed that some sequence segments are in wrong positions of the genome assembly [7]. Therefore the integration of all available chicken mapping resources will be essential for improving the quality of the assembly, building a more reliable and informative resource. In addition to the genetic and BAC contig maps that have already been used, the RH map will thus provide an independent source of data to assist the chicken genome sequence assembly process towards a finished quality sequence.

RH panels are available for several domestic animals: cow [8], pig [9], horse [10], dog [11] and cat [12]. The successful production of a RH panel in chicken is quite recent [13] and therefore only a limited number of chicken chromosomes have been studied to date, namely: GGA4 [14], GGA5 [15], GGA7 [16], GGA14 [7], and GGA15 [17].

We present here the radiation hybrid map of chicken chromosome 2, built by using markers chosen from the GGA2 genetic map and a substantial number of additional markers developed from chicken EST data. Markers from the genetic map are essential to anchor the RH map onto GGA2. Markers developed from EST data were chosen on the basis of existing comparative mapping information data, indicating conservation of synteny with HSA1, HSA3, HSA6, HSA7, HSA8, HSA9, HSA10, HSA18 and HSA22. They were used primarily to saturate the map in markers and also to increase the precision of comparative maps. The GGA2 RH map obtained was aligned with the genomic sequence assembly to detect eventual discordances.

## Results and discussion

### Development of EST markers

Eighty eight microsatellite markers and 10 genes were selected from available data in the literature or the databases. In addition to this, 219 markers were developed from EST data to saturate the map. At the time this study was initiated, comparative mapping data suggested conservation of synteny of GGA2 with 9 human chromosomes and therefore genes were selected from regions of HSA1 (9 markers), HSA3 (28 markers), HSA6 (22 markers), HSA7 (10 markers), HSA8 (84 markers), HSA9 (19 markers), HSA10 (7 markers), HSA18 (35 markers), HSA22 (5 markers). Primers were developed using the Iccare web server [18] with the constraints specific to RH mapping. One hundred and seventy eight out of 219 markers (81 %) amplified successfully. This high success in primer design using EST is comparable to what was achieved in other studies using the Iccare web server [16,19,20].

### GGA2 RH map (figure 1)

**Figure 1:**
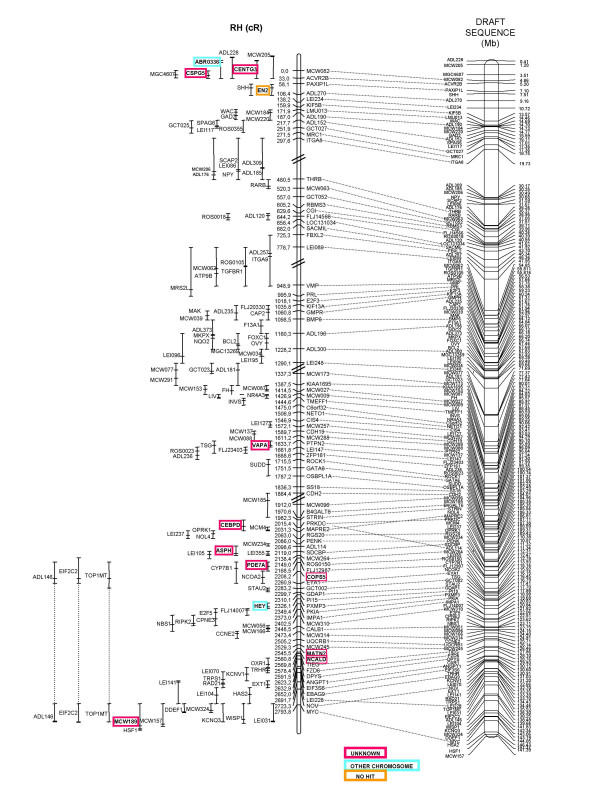
**Radiation hybrid map of chicken chromosome 2 and comparison the draft sequence assembly**. The GGA2 RH map (left) is 2794 cR long and is aligned to the genome sequence assembly (right). The limits between the 5 framework groups of the RH map are indicated by double slashes. Markers present only in the comprehensive map are indicated with their most likely position and the confidence interval, to the left of the map. The coloured framed boxes indicate the results from the BLAST analysis, for the markers that were not found on the chromosome 2 sequence assembly .

Altogether, genotyping data were obtained for a total of 270 markers. Two-point analysis with CarthaGene using a LOD threshold of 4 enabled the construction of 5 GGA2 linkage groups containing a total of 198 markers, including all the microsatellite and gene markers from the genetic map. Out of the remaining markers, two (*LOC51059 *and *ALDH5A1*) should have been integrated into the GGA2 RH map, given their location within the sequence assembly. One explanation, concerning the lack of these 2 markers, could be that the retention frequency, that is unusually low for these two markers and indicative of PCR problems, did not allow attaining a LOD score value significant for linkage. The other 70 markers map either to other chromosomes or to unknown regions. They correspond to the external boundaries of the regions of conserved synteny with human, from which EST were chosen for marker development.

The final RH map of chicken chromosome 2 is 2794 cR_6000 _long and comprises 86 markers. A one-to-one comparison with the genetic map shows a good overall agreement, with a few improvements over the genetic map; for example markers *ADL114 *and *MCW310 *are mapped with a higher precision than previously on the GGA2 linkage group. At the end of the chromosome (position 2794 cR), the 3 markers (*MCW157*, *MCW189*, and *MCW0073 */ *HSF1*) were in the same order in the linkage group, but its orientation is different from the genetic map. These markers are mapped on the comprehensive RH map (less significant than the framework one), so the orientation given in the genetic map may be the right one.

The corresponding comprehensive map comprises a total of 198 markers (figure 1).

### Comparative maps (figure 2)

**Figure 2:**
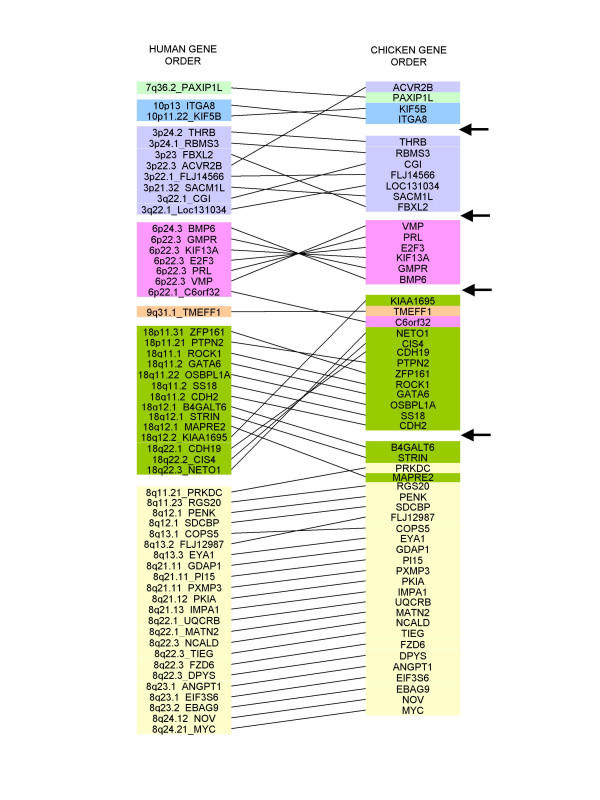
**Comparison of gene orders between the GGA2 RH map and the homologous human regions. **The GGA2 RH map (this study) is compared to the order of homologous genes in human (left) Each colour (plain boxes) corresponds to a human chromosome. Arrows on the right indicate the gaps between the 5 framework maps.

As a result of our map construction strategy, based in great part on the development of EST markers, we found a few observations worthy of comment.

Based on the information that the microsatellite *MCW0189*, identified as being in the *LIMK2 *gene, mapped to the GGA2 genetic linkage group[21], 5 chicken EST markers were developed, corresponding to 8 Mb of HSA22q12.2 containing the homologous gene. Recently, Jennen et al indicated that the mapping of *LIMK2 *was erroneous and that it was located on the GGA15 RH map [17]. We confirm here this result, as all of our five markers mapped to the GGA15 RH linkage group (data not shown). The *MCW0189 *marker remains on the GGA2 RH map, at the position corresponding to where the former *LIMK2 *would have been expected. The result of the BLAST search of *MCW0189 *in the genome assembly indicated a homology with a sequence fragment of unknown location. No similarity with a *LIMK *sequence could be found in this fragment.

A recently published paper describing the mapping of *IL1B *on GGA2 suggested a conservation of synteny with HSA2q [22]. The *IL1B *primers we tested failed to amplify correctly, but other chicken EST markers located near *IL1B*, whose position is 113.3 Mb on HSA2q, had been used in another mapping project in our laboratory [16]. Of these, *ACTR*, positioned at 111.9 Mb and *BIN1 *at 125.1 Mb on HSA2, were mapped to GGA7. If *IL1B*, located between these two markers in human, is indeed on GGA2, the fragment of conserved synteny must be very small. The sequence of *IL1B *in the chicken genome assembly is in a fraction of unknown location.

Previous data suggested that the *RYR2 *gene, mapped on HSA1, was located on GGA2 [23]. We have therefore tested 9 chicken EST markers orthologous to HSA1 genes, but only one (*FH*) could be localized on the GGA2 RH map. As this localisation could be questioned, the identity of the fragment was confirmed after sequencing of the PCR product.

As already observed for other chicken chromosomes [7,15-17,24-26], the GGA2 regions investigated here, corresponding to regions of human chromosomes 3, 6, 7, 8, 9, 10 and 18 showed a high number of intra-chromosomal rearrangements within the regions of conserved synteny.

### Alignment of the RH map to the genomic sequence (figure 1)

A first draft chicken genome assembly was recently deposited into public databases by a team led by R. Wilson and W. Warren, from the Washington University School of Medicine in St. Louis (1st March, 2004, ). The sequence coverage is 6.6X. For GGA2, 147.590.765 bp were sequenced, 166 known genes were identified, and a total of 1432 genes were defined altogether when including results from prediction programs.

For each gene or microsatellite marker from our GGA2 RH map, we compared the fragment sequence with the chicken genome sequence assembly by using the BLAST algorithm [27]. The position for each marker in the sequence is indicated in figure 1. The agreement between the RH map and the sequence order is almost perfect, with only a few local inversions that can be put on the account of genotyping errors or other artefacts that may remain despite all precautions taken, such as the double genotyping process.

In the upper part of the chromosome, several markers absent in the sequence assembly could be localized on the RH map: sequences corresponding to *CENTG3 *and *CSPG5 *genes are aligned with "Unknown" sequence (not assigned to known chromosomes) and *ABR0336 *is found on the GGA27 sequence. The RH two point LOD scores between *CENTG3*, *CSPG5*, *ABR0336 *and their neighbours on GGA2 (*MCW082*, *ACVR2B *or *PAXIP1L*) are higher than 6. In addition, the genetic map confirms the localization of *ABR0336 *on chicken chromosome 2. In the same region of the RH map, *MCW071 *(a microsatellite in the *EN2 *gene) belongs to a genomic region with no sequence available (no blast hit); the genetic map confirms the RH position of this marker. These results suggest possible improvements to be made in the sequence assembly for this region of chromosome 2.

On the sequence assembly, the *TSG *gene, at position 116.4 Mb, is close to *EYA1 *and *STAU2*, whereas on the RH map *TSG *maps at 1660 cR, close to *PTPN2 *and *LEI0147*, located at positions 96.4 and 97.3 Mb respectively. At the same position as *TSG *on the RH map, the *VAPA *gene corresponds to a sequence fragment of unknown location. Moreover, the RH map in this region is in agreement with comparative mapping, so we suspect there may be problems in the sequence assembly in the *TSG *region.

Around position 2100 cR, two local inversions between the RH map and the sequence orders are observed. The first concerns *PRKDC *and *MAPRE2*, and the second *SDCBP *and *ADL114*. In both cases, EST markers mapped close to these genes are missing in the GGA2 sequence assembly: *CEBPD *near *PRKDC *and *ASPH *near *SDCBP *are in the fraction of unassigned sequence. However, in the case of the *PRKDC*-*MAPRE2 *region, the sequence assembly is in agreement with the gene order on human chromosome 8. The order of the markers in the RH map is supported in both cases by a difference of LOD greater than 8, when compared to the alternate order.

In the lower part of the chromosome, several markers localized on the RH map are absent from the GGA2 sequence assembly: the *HEY *gene at position 2330 cR is localized on GGA26 in the assembly, whereas the two-point LOD scores with its neighbours on the GGA2 RH map are trust-worthy (11.5 with *IMPA1*, 8.8 with *PKIA*). The *MATN2 *and *NCALD *genes are in the unknown fraction of genomic sequence, whereas the 2-point LOD scores with their neighbours, around 2550 cR, are higher than 6. At the end of the chromosome (position 2690 cR), we observed an inversion between the RH map and the sequence concerning the *NOV *gene and the microsatellite *LEI228*. The order in the RH map is supported by a difference of LOD greater than 4 when compared to the order in the sequence assembly.

Altogether, out of 198 markers localized on GGA2 through RH mapping, only one, labelled "no hit" in figure 1, could not be found by BLAST in the chicken genome sequence; 10 were assigned to existing genomic sequence of unknown location; 2 were assigned to a wrong chromosome and 4 were not localized at the same position when both maps were compared. With less than 10% of markers either missing in the sequence or for which the two maps disagree, these results confirm the high quality of the genome assembly, with only few finishing improvements to be made in the near future.

The GGA2 RH map is available on the ChickRH web server  also used for RH genotyping data collection.

## Conclusions

We have built a high resolution radiation hybrid map of chicken chromosome 2 using the chickRH6 panel. Our goal was to provide jointly a source of potential polymorphic markers and of candidate genes for QTL mapping on this chromosome. In the course of our work, the first draft chicken genome assembly was released and we aligned it to our GGA2 RH map. Although the sequence assembly is globally in good agreement with our data, a limited number of discrepancies and the mapping of sequence fragments of unknown location or of markers not present in the genomic sequence, show that RH mapping it still useful.

Future developments of the chicken RH map will now be based on the genomic sequence, using it for choosing STS markers in selected regions to develop RH framework maps and thereby detect eventual problems in the genomic sequence assembly. This is clearly needed in the regions for which the genetic map is still not complete, such as some microchromosomes, but also for parts of macrochromosomes, as shown in this study.

## Methods

### Radiation hybrids

The generation of the 6000 rads chicken RH panel has already been described [13]. This panel, named ChickRH6, consists of a total of 90 hybrids. The mean retention frequency of markers is 21.9%.

### Gene selection and primers design

Seventy-seven microsatellite markers well distributed along chicken chromosome 2 were selected from the published chicken genetic map. In addition, 11 other microsatellite markers and 10 genes were selected from other published data [28]. Primer information for these markers can be found in the ARKdb farm animal database  and the ChickAce database .

Primers for 219 EST were designed using the Iccare web server [18], . All publicly available chicken EST (> 420,000) were collected in a local database and compared to the human genome sequence. EST selection targeted towards GGA2 was based on available comparative mapping data with the human genome. Long introns, whose positions could be predicted in chicken on the basis of their position in the human genes, were avoided and primers were chosen in the most divergent regions of the chicken/human sequence alignments, to avoid cross-amplification of the hamster DNA present in the hybrids. Information on primers for EST markers is given in the additional table.

### PCR conditions

PCR were carried out in 15 μl reactions containing 25 ng hybrid DNA, 2 mM MgCl_2 _(Life technologies: Carlsblad, CA, USA), 0.3 U Taq DNA polymerase (Life technologies), 1X buffer (Life technologies), 200 μM of each dNTPs (Life technologies), 0.25 μM of each primer, and 1X loading buffer (350 mM sucrose and 0.2 mM cresol). After a 5 min denaturation step, 30 to 35 cycles (depending on the primers) of 30 sec at 94°C, 30 sec at annealing temperature, 30 sec at 72°C, were performed, followed by a final elongation step of 5 min at 72°C. Chicken DNA was used as positive control; hamster DNA and TE were used as negative controls.

PCR amplification results were scored on 2% agarose gels. Each marker was genotyped twice.

### Map construction

The CarthaGene program [29] was used to build the RH map for chromosome 2 . First, linkage groups were constituted by using a two-point LOD threshold of 4, after which a 1000:1 framework map (a map whose likelihood is at least 1000 fold higher than the next possible highest likelihood using the same markers in alternate orders) for each group was built under a haploid model. Then, the framework maps obtained were aligned on the genetic map and the orientation and distance between each group were estimated using the "printbestmap" option in the Carthagene program. Finally, after this final validation, all distances between markers were re-evaluated under a diploid model.

Markers not included in the framework map, but displaying two point Lod scores higher than 4 with framework markers, were mapped relative to the framework map by using the CarthaGene "buildfw" option which calculates the most likely position of a marker and gives a confidence interval.

The map was drawn using the MapChart software [30].

### Maps comparison

Data on the location of the chicken genes in the sequence assembly was obtained by BLASTN searches using the Ensembl  browser.

Data on human gene order were obtained from the Iccare web server.

## Authors' contributions

SL, MD, SB, FV and KF carried out the molecular study. SL built the RH map, and drafted the manuscript with FP. MM made the ChickRH6 panel. AV coordinated the project and finalized the manuscript.

## Supplementary Material

Additional File 1 **Description of the markers**. The GCTXXXX markers were developed using the Iccare web server. The EXTXXXX markers were chosen from published information. Click here for file
